# Advances in gene transfer technologies: comparing viral and non-viral vectors for therapeutic applications

**DOI:** 10.1007/s13205-026-05016-2

**Published:** 2026-08-21

**Authors:** Praveen Halagali, Raagul Seenivasan, Amatha Sreedevi, Virendra S. Ligade, Parashuram Bugadannavar, Himanshu Sharma, Sachchida Nand Rai, Vamshi Krishna Tippavajhala

**Affiliations:** 1 Department of Pharmaceutics, Manipal College of Pharmaceutical Sciences, Manipal Academy of Higher Education, Manipal, India; 2 Department of Pharmaceutical Regulatory Affairs and Management, Manipal College of Pharmaceutical Sciences, Manipal Academy of Higher Education, Manipal, India; 3 Department of Pharmacy Practice, Manipal College of Pharmaceutical Sciences, Manipal Academy of Higher Education, Manipal, India; 4 Department of Pharmacology, Nims Institute of Pharmacy, Nims University Rajasthan, Jaipur, 303121 India; 5 Saveetha Institute of Basic Medical Sciences (SIBMS), Saveetha Institute of Medical and Technical Sciences (SIMATS), Saveetha University, Thandalam, Chennai, 602105 Tamil Nadu India

**Keywords:** Non-viral vectors, Gene transfer, Genetic engineering, Ex vivo gene transfer, In vivo gene transfer, Viral vectors

## Abstract

Gene and cell therapies have emerged as transformative approaches for treating a wide range of genetic and acquired diseases. Central to their success is the development of safe and effective gene delivery systems, categorized broadly into viral and non-viral vectors. Each system has its own advantages and limitations, requiring careful consideration of the target tissue, disease, and the balance between safety, efficacy, and scalability. Viral vectors, including adeno-associated viruses, retroviruses, lentiviruses, herpes simplex viruses, and adenoviruses, offer high transduction efficiency and specificity but raise concerns about immunogenicity and production challenges. Non-viral systems, such as lipid nanoparticles (LNPs) and other synthetic carriers, provide scalable, cost-effective, and potentially safer alternatives but often face hurdles in transduction efficiency and targeted delivery. This review provides a comprehensive overview of the current status of these vector systems for in vivo and ex vivo applications. Key comparisons are made across safety, efficacy, scalability, and immune responses, highlighting recent advancements and innovative approaches. We also discuss the outlook for next-generation gene transfer technologies, focusing on improvements in vector design, manufacturing, and application versatility. By addressing these considerations, we aim to inform the development of optimized therapeutic strategies that leverage the unique strengths of each delivery system.

## Introduction

The advancement of biomedical technology has enabled the medical sector to make tremendous progress in the prevention, diagnosis, and treatment of various life-threatening diseases, especially those caused by genetic abnormalities. Nevertheless, several disease-related conditions cannot be treated with traditional pharmacological agents, biologics, or peptide-based therapeutics (Valeur et al. 2017). Many conditions appear at birth or in old age, and the usual drugs are not used to treat the resulting genetic alterations. As novel therapy modalities continue to evolve, treatments are becoming disease-symptom oriented and are also directed at the genetic causative factors. One such effective therapy is gene therapy, which aims to treat the disease at the genetic level by correcting defective or altered genes that cause the disease. The disorder may be highly diverse, depending on the nature of the genetic mutation, deletion, or insertion, and impacting normal metabolic activities, the cell cycle, the cytoskeleton, ligand-receptor interactions, and extracellular protein activities (Balakrishnan et al. 2024; Mehnath 2026). Gene therapy may be the choice that offers hope and, in certain cases, the only possible cure (Cevher et al. 2012). The overall significance of gene therapy lies in its ability to modify, replace, delete, or repair genes to produce a therapeutic effect (Gascón et al. 2013). The foundation of genetics was first established by Gregor Johann Mendel, who conducted a study of inheritance and proved that traits are inherited as units, later called genes. The material component of these genetic units was clarified in 1953, when Francis Crick and James Watson proposed that these units of inheritance had a molecular structure: double-stranded Deoxyribonucleic Acid (DNA), a double helix. The second molecular biology breakthrough was the discovery of restriction nucleases in 1970, which enabled genetic engineering and the creation of the therapeutic field of reverse genetics (Gonçalves and Paiva 2017).

**Fig. 1:**
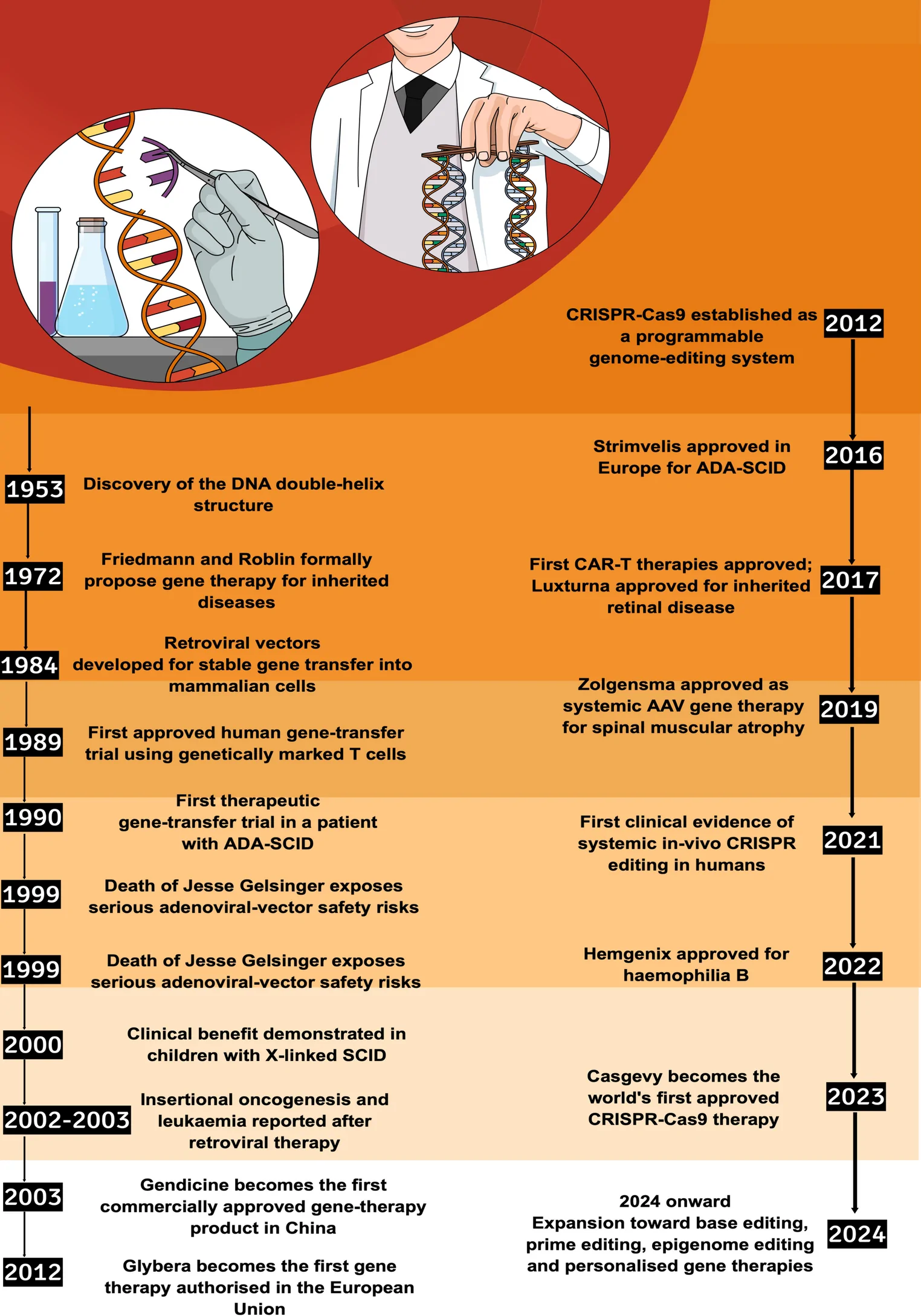
Evolution of gene therapy from conceptual development to precision genome editing (Prepared By Using Mind the Graph)

Over the past decades, the field of gene therapy has become of utmost importance in molecular medicine, with vast prospects for treating and possibly curing inherited and acquired genetic diseases in humans. Evolution of gene therapy from conceptual development to precision genome editing is depicted in Fig. 1. The timeline highlights major scientific discoveries, first-in-human studies, safety setbacks, regulatory approvals, and the transition from viral gene addition to CRISPR-based genome editing. Recent advancements in recombinant DNA technology have increased its medical use in the 21st century, especially in prenatal genetic screening and genetic counselling (Annas and Elias 1990; Flake 2003). Individuals with pathogenic mutations who are diagnosed and are likely to develop the condition through genetic screening are the most suitable candidates for gene therapy-based clinical interventions. The clinical use of gene therapy has grown widely over the last several years due to advances in science and the development of new technologies (Verma et al. 2000). Gene therapy is clinically effective in correcting genetic defects that cause inherited diseases, e.g., haemophilia, cystic fibrosis, thalassemia, hypercholesterolemia, and RPE65-mutation-induced retinal diseases, including Leber congenital amaurosis and retinal dystrophy (Govindasamy et al. 2025; Saha et al. 2025). Gene therapy (GT) has also been among the common research areas in the management of acquired diseases such as Alzheimer’s disease (AD), cancer, Parkinson’s disease (PD), and AIDS (Hanania et al. 1995; Seroogy and Fathman 2000; Shimamura et al. 2013). Even though gene therapy has therapeutic potential, its ethical and regulatory concerns, particularly the alteration of germ-line genes, are significant. Germline genetic modification is heritable and can affect future generations; therefore, there is a need to ensure strict control and regulation to ensure ethical conduct. Germline GT has thus been linked to serious ethical, legal, and social issues that today limit its clinical application (Karpati and Lochmüller 1997; Savulescu 2001).

### Overview of gene therapies

General-type GT can be divided into three approaches: gene expression inhibition, artificially increasing gene expression, and direct modification or correction of defective genes. RNInc. RNAi interference is among the most efficient ways to silence gene expression, in which gene expression is inhibited by inhibiting mRNA translation. RNAi modalities can be delivered into cells to silence mRNA molecules using small interfering RNAs (siRNAs), microRNAs (miRNAs), short hairpin RNAs (shRNAs), and antisense oligonucleotides (ASOs), thereby decreasing protein synthesis (Khurana et al. 2019; Xu et al. 2019). On the other hand, the therapeutic modalities in gene overexpression or gene replacement therapy involve a functional copy of a gene, usually in the form of plasmid DNA (deoxyribonucleic acid), to induce the cells to express the gene. This strategy relies on the fact that underexpressed proteins are either transient or persistent, depending on whether they are deficient or dysfunctional in disease. Another important mode of application is corrective gene therapy, which uses engineered nucleases to induce targeted mutations in the genome. More specific genome editing is made possible in eukaryotic systems using highly popular programmable nucleases (Gaj et al. 2013; Lehmann et al. 2017; Hong et al. 2019). Some such clinical applications include ex vivo gene therapy, in which primary cells are isolated from the patient, genetically modified, and transplanted back into the patient through autologous transplantation. To ensure gene therapy is effective, the most important aspect is the use of a safe and effective gene delivery mechanism, which delivers therapeutic genetic material into specific cells or tissues. The novelty of gene transfer could thus be estimated as the provision of gene copies into live cells that enable the production of a desired therapeutic protein, with several limitations. The phosphate groups make DNA molecules highly hydrophilic, very large, and negatively charged, so they cannot pass through cell membranes.

**Fig. 2:**
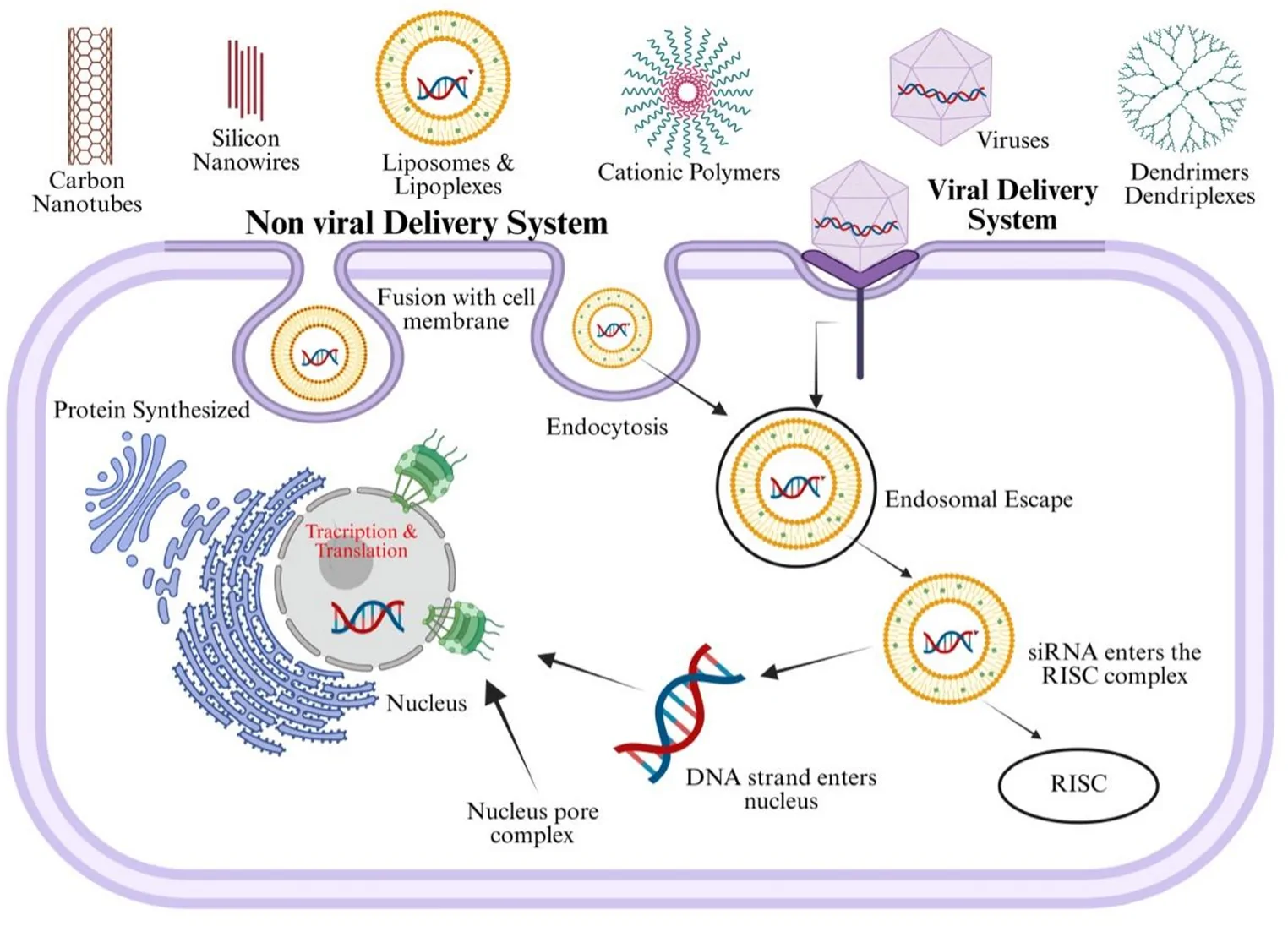
Gene delivery vectors (Created By Using Bio-render)

Additionally, unprotected DNA is rapidly degraded by extracellular and intracellular nucleases. As a result, gene delivery vectors (viral or non-viral) provide safety and protection for the genetic material during its delivery into target cells (Mali 2013; Li et al. 2020). Cellular uptake and cellular functions transfer intracellular cargo to the target compartments in question and, as such, dictate the outcome rates of gene delivery and targeting. Endocytosis is the most prevalent pathway through which the viral and non-viral gene delivery systems access cells. It includes phagocytosis, endocytosis via clathrin, caveolin, and clathrin-independent pathways, caveolae-independent endocytosis, and macropinocytosis (Behzadi et al. 2017). Once endocytosed, the genetic cargo must escape endosomal compartments to perform its desired activity, e.g., silence genes or overexpress a gene. In addition to endocytosis, a nanocarrier system can also gain access to the cell through membrane fusion. The nanoparticles, such as nanoplexes and cubosomes, have intrinsic fusogenic properties and can directly fuse with the cell membrane, increasing cell uptake and intracellular delivery efficiency (Chen et al. 2009; Weng et al. 2015; Dyett et al. 2019). The gene delivery vectors are depicted in Fig. 2.

The types of gene delivery vectors include carbon nanotubes, viral vectors, liposomes, lipoplexes, cationic polymers, dendrimers, dendriplexes, and silicon nanowires. They are vectors that transport therapeutic nucleic acids (e.g., DNA or siRNA) into cells via endocytic pathways or direct cell-membrane fusion. Cellular internalisation enables the release of vectors once they are subjected to endosomal compartments, as they escape into the cytosol and discharge their cargo. Delivered siRNA associates with the RNA-induced silencing complex (RISC) and allows sequence-specific (cytosilencing) of mRNA. Delivered DNA, on the other hand, will enter the nucleus and will be subjected to transcription and translated into the cytoplasm to provide the necessary protein.

### Significance of gene transfer methods

The relevance of transfer methods lies in their ability to improve the practice used in the production of pharmaceutical drugs. The significance of gene transfer methodologies is that they enable the production of pharmaceuticals. One factor that makes gene therapy effective is the ability to deliver large amounts of genes to target cells. Several methods have been developed for intracellular delivery of DNA or RNA; which is the most effective one in a particular case is yet to be identified. The choice of the appropriate gene transfer methodology depends on several factors, including cost, reproducibility, cytotoxicity, delivery systems, ease of application, and transfection efficiency. Based on these parameters, there is a gain in the gene transfer processes for both viral and non-viral methods (Hahn and Scanlan 2010; Ereej et al. 2024). A comparative view of the methods used to transfer genes is given in Table 1.

**Table 1:** Comparative view of the methods used to transfer genes

Type of method	Description	References
Physical methods		
Electroporation	This method increases membrane permeability and injects our desired cargo by using controlled, short electric shocks.	Gräber (1990; Young and Dean 2015; Cervia and Yuan 2018)
Thermal-assisted gene transfer	This method uses exogenous heat to attain the same level of cell penetration at lower voltages.	Bulysheva et al. (2015)
Biolistic	This method entails covering the target tissue with the DNA-particle combination and using a high-power shot to insert the DNA into the tissue, specifically into the cells.	Yang et al. (2004; O’Brien and Lummis 2011; Godbey 2014)
Microinjection	Using microneedles, microinjection is a physical, direct method of delivering genes.	Chow et al. (2016)
Ultrasound-assisted gene transfer	The cavitation caused by ultrasound increases the penetration and delivery of genetic material to cells by causing microbubbles to develop and burst.	Passineau et al. (2010; Tomizawa et al. 2013)
Hydrodynamic gene transfer	Depending on the volume and pace of the injected DNA solution, it creates hydrodynamic pressure that improves the permeability of the capillary endothelium and cargo delivery to the parenchyma surrounding these capillaries.	Suda and Liu (2007)
Magnetofection	In this case, the particles are delivered to the cells via the magnetic field.	Yellepeddi (2015)
Chemical methods		
Calcium phosphate-mediated	DNA, CaCl₂, and phosphate buffer are combined to create a co-precipitate of calcium phosphate and DNA.	Sambrook and Russell (2005)
DEAE-dextran mediated	The negatively charged inorganic phosphate backbone of DNA attracts the positively charged DEAE-dextran electrostatically, creating a complex with a net positive charge that enables interaction with the negatively charged membrane.	Mack et al. (1998)
Cationic lipid-mediated	Positively charged DEAE-dextran electrostatically attracts the negatively charged phosphate backbone of DNA, creating a compound with a net positive charge that permits interaction with the negatively charged membrane.	Felgner et al. (1993; Mortimer et al. 1999; Zhu and Mahato 2010)
Liposomes	The lipids utilized in transfection have a hydrophobic tail and a hydrophilic cationic head. These lipids can adopt a unilamellar arrangement, and their interactions are controlled by the negative charge on the nucleic acid and the relatively positive charge on the lipids	Hug and Sleight (1997)
Polymers	These colloidal drug-delivery vehicles, which take the shape of unilamellar or multilamellar vesicles, can be neutral or charged.	Eliyahu et al. (2005; Barua et al. 2011)
Biological methods		
Adenoviruses (Advs)	Advertisements are well-defined by a size of approximately 26–40 kb, by being non-integrated and non-enveloped, and by being linear DNA viruses enclosed by an icosahedral particle with a measurement of about 950 Å. These are non-integrating viruses. The latest Ads can deliver transgenes up to 36 kb	Roy-Chowdhury and Horwitz (2002; Borovjagin et al. 2014; Kaiser 2020)
Adeno-associated viruses (AAVs)	AAVs are non-enveloped, little less than 3/4-inch viruses that contain linear, single-stranded DNA. The AAV genome is extremely tiny, measuring only about 5 kb. Transgene insertion is limited to 4 kb; the genes in the middle are eliminated, and the remaining space is utilized as an expression cassette that contains a reporter and a gene of interest.	Barzon et al. (2005; Crystal 2014; Naso et al. 2017)
Retroviral and lentiviral vectors	Retroviruses are usually diploid, single-stranded, circular-enveloped RNA viruses with a diameter of 80–120 nm and a genomic size of 7–11 kb. Since the mid-1990s, lentiviruses, a subtype of retrovirus, have been widely employed as gene delivery vectors; a transgene with an 8–10 kb size can be introduced.	Yi et al. (2011; Vargas et al. 2016)
Bactofection	Bactofection is the process of utilizing bacteria to deliver genes to target cells. It involves transferring the transgene-containing plasmid into the target cell together with the modified bacterial strain.	Palffy et al. (2006; Johnson et al. 2019)
Virus-like particles	These are non-genetic material viruses	Petry et al. (2003)
Erythrocyte ghosts	The red blood cells that have lost their cytoplasm and have been extruded into nanoscale spherical bodies are called erythrocyte ghosts	Teorell (1952)
Exosomes	A microvesicle (30–90 nm) is compact and membrane-bound and has an endocytic origin	O’Loughlin et al. (2012)

## Viral vectors

### Adenoviruses

The Adv are vectors, or gene delivery vehicles, developed since the early 1980s, since their original isolation in the 1950s of human adenoid tissue (Berkner 1988). Family Adenoviridae Adenoviridae is a division of viruses formerly subdivided as the genera Aviadenovirus and Mastadenovirus: the former has viruses that infect birds, and the latter viruses that infect mammals. In 2002, two additional genera were described: Atadenovirus, due to the abnormally high proportion of adenine and thymine in the genome of the first known members of the genus (parasites of ruminants, avians, and marsupials), and Siadenovirus, after the putative sialidase homolog encoded by the representatives of the genus (Gnutova 2014). The proportion of guanine and cytosine in the DNA molecules and their red blood cell agglutination capacity are used to group human adenoviruses into 6 species (named A-F). A further sub-classification of the human adenoviruses is into more than 50 serotypes (numbered in Arabic numerals) based on the neutralisation assay (Shenk 2001).

The human Ad2 and 5 Ad5 of species C serve as the primary basis for recombinant adenoviral vectors. These serotypes cause moderate, non-oncogenic respiratory diseases in people. Positive outcomes for the use of Adv vectors in in vivo gene therapy have resulted from their safety, as well as from the fact that adenovirus-based vaccines have been tested in humans without any adverse effects (Kovesdi et al. 1997). Due to their deletion in the E1 region of the viral genome, which makes them replication-deficient and only able to replicate in specially created complementing cell lines, recombinant adenoviral vectors have also boosted safety. Recombinant adenoviral vectors in human serotypes 2 and 5 also have the benefit of producing high-titer vectors (10^13 virus particles/mL), which makes them practical for usage in vivo. Another important attribute of Ad vectors is their stability in the blood, which allows them to deliver the gene when given intravenously. Ad not only infects dividing cells but also postmitotic ones, and it has devised a particularly effective mechanism for transferring its genome into the nucleus. The genome is not integrated into the chromosomes, and therefore, the risk of insertional mutagenesis is reduced. Vectors containing a 7.5-kb deletion are known as first-generation vectors, including the E1-deleted Ad2 and Ad5 vectors. Additional deletions of viral genes may be done to expand the size of these vectors. These properties have raised major concerns about the application of Adv2 and Adv5 as gene delivery vectors, which, in turn, have led to the development of a range of techniques for manipulating these vectors and forming recombinant vectors with relative ease. However, oncolytic Adv2- and Adv5-based vectors used in gene therapy also have several disadvantages. They are not specific to a cell type and have tropism for the viruses they harbour, infecting any cell type that expresses the appropriate receptors.

On the other hand, several cell types that are thought to be beneficial for gene transfer also have poor cellular receptor expression, which makes infection ineffective. The fact that Adv2- and Adv5-based vectors elicit both innate and adaptive immune responses is another significant drawback of their in vivo use. There has been much discussion about how to overcome these drawbacks so that adeno-viral vectors can reach their full potential.

### Retroviruses

**Fig. 3:**
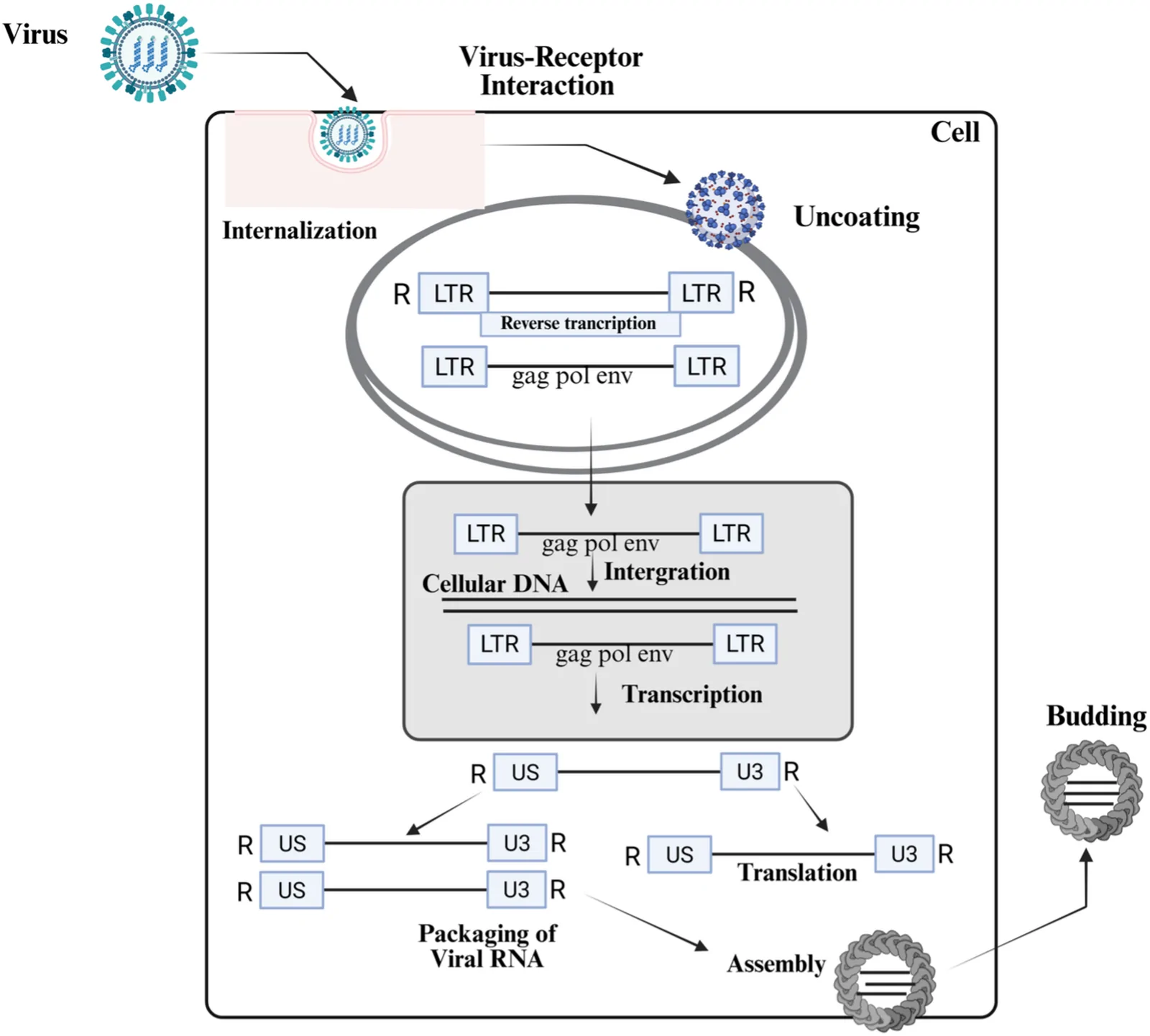
Salient points in the life cycle of a retrovirus (Created By Using Bio-render)

There are various methods for transferring genes, and each utilises different mechanisms. Among them, retroviral vectors have several advantages over earlier gene transfer technologies, especially because their life cycle is well characterised. The genetic material of retroviruses is contained in the form of RNA. This RNA is reverse transcribed into DNA upon entry into the host cell, and the resulting DNA is then inserted into the host genome. This integrated viral DNA, referred to as a provirus, is retained in the host cell (Fig. 3). The other significant benefit of retroviral vectors is that they can insert the gene(s) of interest at low copy number. The latter, in combination with their efficient and biologically stable integration, reduces the risk of gene rearrangement and maintains uniform gene expression. The retroviral vectors currently in use are mostly based on murine or avian retroviruses. The most common is the Moloney murine leukaemia virus (Mo-MuLV), which has been the most used retroviral vector in gene therapy studies in rodents and humans. The popularity of Mo-MuLV-based vectors can be attributed to profound biological knowledge, the ability to produce relatively high titers of recombinant virus, and the presence of retroviral variants that can infect human cells. They are amphotropic and xenotropic retroviruses; however, ecotropic Mo-MuLV only infects cells in rats (see the section on changing the Mo-MuLV infectivity spectrum). The retroviral vector system has two major parts: (i) a recombinant retroviral vector is used that contains the gene(s) of interest and trans-expressing constructs, and (ii) the retroviral structural proteins. All these elements allow the synthesis of viral recombinant particles that can effectively infect host cells and transfer the desired genetic material (Salmons and Gunzburg 1993).

### Lentiviruses

They are amphotropic and xenotropic retroviruses; however, ecotropic Mo-MuLV only infects cells in rats (see the section on changing the Mo-MuLV infectivity spectrum) (Bukrinsky et al. 1993). The property is beneficial for numerous gene-therapeutic protocols targeting post-mitotic, highly differentiated cells. These lentivectors are now used in approximately 1.4% of clinical trials (Huh 2025). The key disadvantage of using g-retroviral vectors in human GT has been insertional mutagenesis. In animal models and cellular systems susceptible to oncogenesis, lentiviruses cause insertional mutagenesis (Bokhoven et al. 2009), although, in general, they are less mutagenic than their oncoretrovirus counterparts (Hematti et al. 2004; Montini et al. 2009). This was soon followed by a positive study in which no abnormal cell growth or enrichment of integration sites around proto-oncogenes was observed to date in the first approved human clinical trial using lentivectors for the treatment of HIV (Manilla et al. 2005; Themis et al. 2006).

### Adeno-associated viruses (AAVs)

Adeno-Associated Virus (AAV) gene therapy is a revolutionary technology for in vivo gene delivery, with a proven track record of safety and efficacy across numerous preclinical and clinical trials. The following is a thorough summary describing the current knowledge, obstacles, innovations, and clinical applications of AAV-based gene therapy. AAV vectors have shown impressive efficacy in gene transfer into tissues, with stable, long-lasting results that persist for many years after a single dose. In particular, this long-lasting efficacy is particularly useful in gene therapy targeting the liver in patients with haemophilia, as well as in metabolic disorders and conditions in which transduction of hepatocytes results in persistent production of therapeutic proteins (Piccolo and Brunetti-Pierri 2025). AAV gene therapy is also used to treat both rare and common skeletal diseases. Approaches applied include gene addition, gene replacement, gene silencing, and gene editing via AAV vectors, which can be effective for numerous disorders such as fibrodysplasia ossificans progressiva, osteogenesis imperfecta, osteoporosis, and osteoarthritis. Research studies confirm both the effectiveness of this kind of treatment and the safety profile, demonstrating low immunogenicity and pathogenicity in skeletal tissues (Lin et al. 2024).

In gene therapy applications, AAV vectors are particularly useful for specific ocular treatments, especially retinal diseases. As the eye is an immune-privileged region, AAV therapy offers the advantage of eliciting a minimal immune response, allowing long-term transgene expression and treatment of both inherited and acquired retinal diseases. Various innovations are being developed to address the limitations imposed by AAV, such as transduction inefficiency and low packaging capacity. For example, it is believed that the development of multivector systems and capsid modifications will enhance AAV therapy and reduce the risk of complications such as uveitis (Huang et al. 2025). Furthermore, the significance of AAV vectors is closely linked to gene-editing techniques. The combination of CRISPR-Cas9 technology with AAV delivery systems allows for precise genome editing in vitro. With the introduction of smaller Cas9 variants compatible with AAV and of optimized promoters and serotypes, the effectiveness and precision of genome editing have increased. However, it is worth noting that certain problems remain regarding off-target effects, the immunogenicity of components involved in the CRISPR process, and the toxicity of the delivery vehicle (Lau and Suh 2017). Despite notable advancements in AAV gene therapy, there are still major immune-related challenges that compromise both effectiveness and safety. Pre-existing immunity against AAV capsid-based vectors is widespread, and it can neutralise vector particles, leading to limited transduction. The activation of innate immunity (e.g., via the complement pathway or Toll-like receptors) can cause infusion-related side effects in patients, ranging from minor symptoms resembling flu-like illness to severe cases of anaphylaxis. Infusion-related problems can increase when administering high vector doses or when there is prior exposure to AAV vectors (Notarte et al. 2023).

Presently, options for managing infusion-related symptoms are limited because there are no universal treatments or diagnostic protocols. While therapeutic approaches such as the use of steroids, the process of plasmapheresis, and other supportive treatments, including the use of antihistamines, exist, there is no consensus on which treatment or a combination of therapies is optimal. Thus, it is clear that more research is needed to identify immune modulation methods that will make the therapeutic process safer for patients (Notarte et al. 2023). Regulatory T cells (Tregs) are a vital cell population in modulating the immune response to AAV therapy. Treg functionality could be improved by using selective immunosuppressants, ex vivo Treg expansion, or receptor modification, leading to possible effective tolerance against vector capsids and transgene products. Treg harvesting might help minimise cytotoxic immune responses that compromise the efficacy of gene therapy (Muñoz-Melero and Biswas 2024). Apart from immunology, AAV vector properties influence genotoxicity. AAV is generally considered a safe vector, but studies indicate that AAV integration might lead mice to develop hepatocellular carcinoma by integrating into loci such as the Rian locus. Factors such as vector dose, choice of enhancer/promoter elements, and timing of administration greatly influence genotoxicity (Chandler et al. 2015).

Important considerations in clinical pharmacology, which are relevant to the therapy of genes using recombinant AAV, are biodistribution, dose-exposure-response profiles, and safety profiles based on immunology. To choose the proper dose and reduce risks when organising trials, it is essential to understand transduction efficiency variability, immune responses, and the durability of gene expression. Also, pharmacodynamics and pharmacokinetics can take into consideration some patient-specific factors and vector product features (Sun and Liao 2022). The strategies under discussion are highly advanced and intended to enhance AAV gene therapy operations. One such strategy is hyperthermia-assisted delivery. Hereby, mild heating increases blood flow and tissue permeability, facilitating the entry of a vector into the body. Moreover, heat regulators can influence gene expression. However, there are also some obstacles, such as thermal dose control, particle stability, and immune reactions (Lin et al. 2026).

**Fig. 4:**
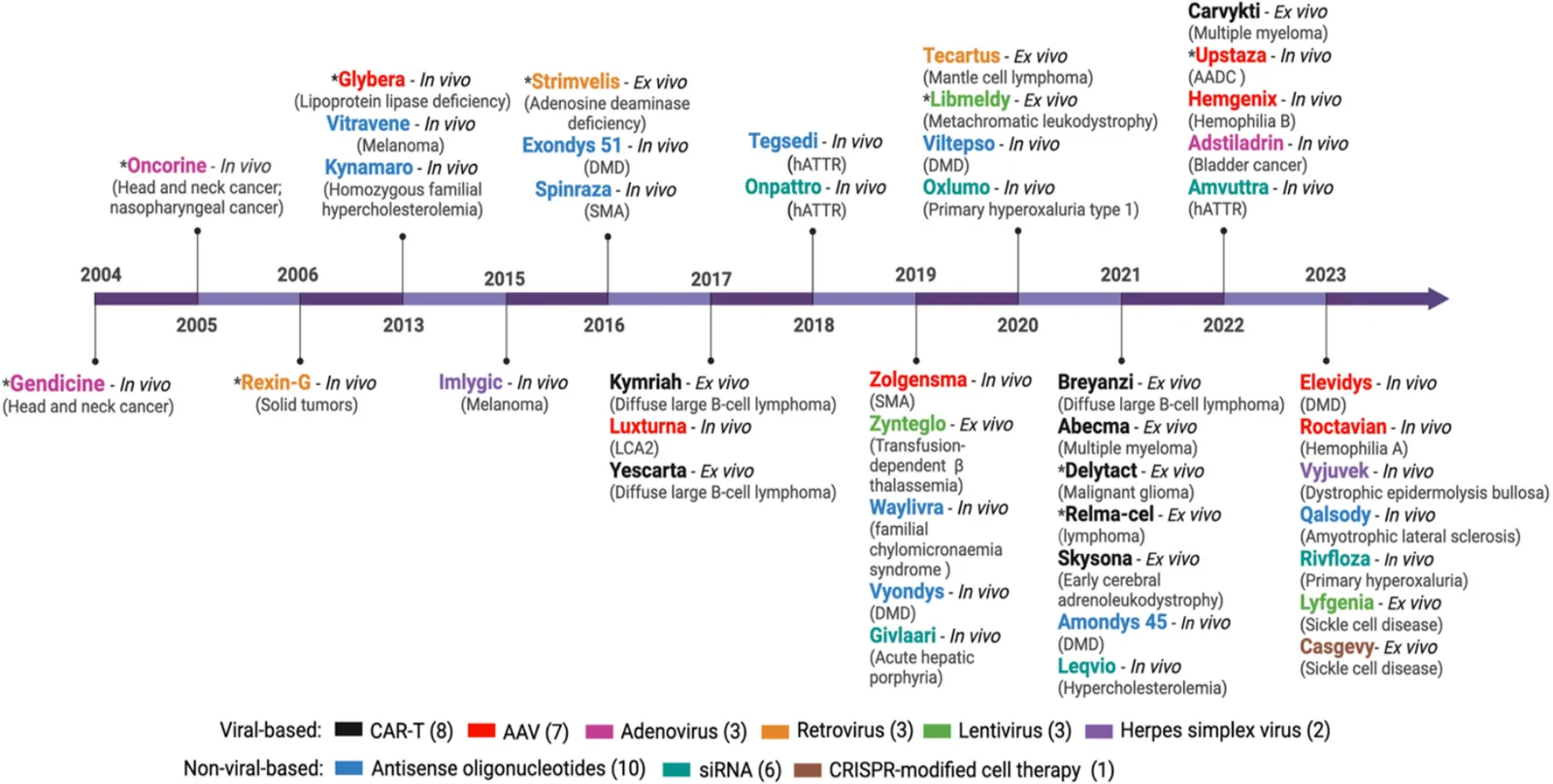
Approved gene therapy products and delivery platforms Reproduced With Permission from (Wang et al. 2024)

Furthermore, the advancement of AAV-based therapies has evolved from gene enhancement to advanced gene editing and somatic gene alteration methods. Regulatory approvals of AAV products, including Glybera, Luxturna, and Zolgensma, have demonstrated progress toward clinical applications, while ongoing studies are pushing the limits of more sophisticated genome-editing approaches using AAV vectors. These strategies offer prospects for the final cure of incurable illnesses; however, they pose difficulties associated with toxicity, immune responses, and delivery efficacy (He et al. 2021). In conclusion, AAV gene therapy is becoming a foundation of modern gene medicine, both because of its long-term genetic instructions and manageable immunogenicity, and because of its increasing clinical applications, ranging from liver disorders and muscle problems to ocular issues and genome editing. Although immune-mediated toxicity, vector toxicity, and optimal dosage remain major challenges, the field continues to develop due to the emergence of new technologies in vector design, immune modulation, and enhanced drug delivery. Approved gene therapy products and delivery platforms are given in Fig. 4.

It has taken quite some time for gene therapy (GT) in the clinical setting to address both the apprehension of others about safety and legitimate concerns about the effects of GT on human disease. At this point, it is clear that there has been sufficient proof of concept for the clinical utility of GT. Interestingly, there are a couple of pioneering programs in GT focused on disorders of the nervous system and the use of adeno-associated virus (AAV) for delivery. In 2017, the first AAV product, Luxturna (voretigene neparvovec-rzyl), was approved for commercial availability; it consists of AAV carrying a cDNA for RPE65 that can be administered via subretinal injection, and is indicated for individuals with biallelic RPE65 mutation-related retinal dystrophy, which could either cause them to suffer vision loss or lead to complete blindness (Russell et al. 2017).

As of November 13, 2018, there were 145 registered rAAV interventional clinical studies. Two vectorized AAV serotypes, AAV1 and AAV2, have received regulatory approval for use in patients as of 2019. Less than a dozen capsid serotypes are currently in clinical trials as vectors, with the most numerous based on AAV2. Nevertheless, more efficient and modern capsids are being used in trials, including newer ones such as AAV8, AAV9, and AAAVrh. 0. The treatment plan in most cases is the targeting of a recessive disease that is monogenic through gene replacement. In small-scale genetic diseases affecting a limited number of patients, Phase I/II trials are often conducted to accelerate product development.

### Herpes simplex viruses (HSVs)

HSV infections may induce recurring orofacial, corneal, anogenital, or any other lesions. Newborn diseases may result in outbreaks and devastating neurological effects. Genital HSV-2 infection leads to the enhanced risk of contracting HIV and is an important factor in the widespread HIV infection around the globe (Schiffer and Corey 2009). The current antiviral therapy can cure acute attacks and outbreaks, but does not cure the infection (Mertz et al. 1988; Wald et al. 1996; Gupta et al. 2004; Corey et al. 2004; Fife et al. 2006). The prevalence of these outbreaks is explained by the fact that HSV establishes latency in the ganglionic neurons of the affected regions. The latent HSV in the ganglia is immune to the effects of traditional antivirals, which is why they are not effective in curing the virus; reactivations often return to normal after therapy is withdrawn. Among these prospective, possibly curative methods is the gene editing specific to latent HSV (Aubert et al. 2016). A recent article used a meganuclease mediated by AAV to eliminate over 90 per cent of the HSV-1 genomes in the superior cervical ganglia of latently infected mice (Aubert et al. 2020). Even though this is a substantial reduction in ganglionic HSV loads after gene editing, it is uncertain how this reduction would apply to human HSV infection. The issue of ganglionic viral loads is usually not the primary concern in an infected person, but rather the symptomatic illness and/or viral shedding and the possibility of infecting another person (Oseso et al. 2016). Current mouse models do not fully resolve the problem: HSV rarely reactivates spontaneously or spreads to peripheral tissues in latently infected mice. The researchers showed that gene editing modifies ganglionic viral loads and the viral shedding generated by small-molecule stimulation of HSV-1 reactivation and peripheral shedding in latently infected mice. Improvement of the therapeutic regimen through simplification, lower doses, and targeted expression of the meganuclease in the liver and ganglia leads to almost complete elimination of unwanted effects in these tissues, which is favourable to further clinical development of the strategy.

HSV-1 is an enveloped virus that infects nerve tissue, and is one of the most common types of infection among humans. It infects approximately two-thirds of the world’s population and causes two distinct forms of infection. Productive or lytic infection occurs in epithelial cells (the outer layers of cells that cover your skin and line most organs in your body). Latent persistent infections occur in sensory neurons; however, the virus can also exist latently in autonomic nervous system neurons (Steiner and Kennedy 1995; Knipe et al. 2021). In addition to existing latently, productive infections can occur in sensory neurons when they reactivate from latency. Researchers use two different forms of HSV-1 vectors in gene therapy (GT) for inherited and acquired diseases: non-replicative HSV-1 vectors and replicating, selectively attenuated vectors. Non-replicative HSV-1 vectors have specific defects that prevent them from being virulent and are primarily used for the treatment of inherited or acquired diseases. In contrast, selectively attenuated oncolytic viruses are commonly used in cancer virotherapy (Manservigi et al. 2010). Both types of vectors are non-integrating vectors, and they have already been used in clinical settings. Further, non-replicative HSV-1 vectors can be classified into two categories: non-replicative HSV-1 genomic vectors (nrHSV-1), which have a live attenuated viral genome, and amplicons. Amplicons have had their lytic genes deleted from their genome and hence are relying on assistance from another virus to be replicated (Lundstrom 2018).

### Advantages and limitations

Viral vectors play a pivotal role in GT and are used to support new therapies across a broad spectrum of medical practices. The tools are potent vectors that utilise innovative methods, like CRISPR, which is the future of medicine. It is on this foundation that various preclinical and clinical studies are being conducted to expand the already remarkable range of VV applications and to improve in vitro production techniques. The importance can also be justified by the fact that almost 70 per cent of gene therapy clinical trials conducted to date have used VV. This number is even more remarkable given nearly 3000 trials. It attracts the special attention of biotechnology and regulatory agencies, including the FDA, to virus-based vector systems (Lundstrom 2018). Among the advantages of VV in GT are (a) in vivo gene therapy, which involves the direct delivery of VV into the patient’s body, providing long-term therapeutic effects. Method: Producing favourable effects, e.g., applied to the nervous system or the liver. VV in cancer therapy VV. Cancer therapy with VV can provide cancer vaccines and oncolytic viruses that infect cancer cells directly, killing them while sparing normal tissues. In addition, they can specifically target malignancies, thereby boosting the body’s immune response against cancer, providing an accurate and efficient way to treat various cancers (Bezeljak 2022). (c). GT for immune disorders: Gene therapy is used to treat immune system disorders. Such treatments have also opened new possibilities for treating diseases like leukaemia, which involve boosting the body’s immune system. VVs in T cells help kill cancerous cells. Genetic disorders: The genetic modification of genetic diseases like cystic fibrosis and muscular dystrophy has a lot of potential, which the viral vectors can offer immensely. They transfer functional genes to neutralise genetic mutations, and the door to better patient outcomes is open.

VVs have transformed the field of gene therapy, but they also pose challenges and disadvantages. These are the pitfalls to consider when seeking safe and effective therapy. The virus vectors used to transfer the genes include (a) Immunogenicity: There is an issue of immunogenicity whereby the immunogenicity of the VVs might cause the body to recognise the VVs as pathogens, leading to an immune response. This will decrease vector activity and may lead to unwanted vector insertion into the host genome, with detrimental consequences. (b) Insertional mutagenesis: Under unintended circumstances, the VVs can enter the host genome, leading to insertional mutagenesis. Such a disruption of current circumstances is not predictable and can even be harmful, especially with long-term treatment. (c) Toxicity: VVs are toxic in host cells, resulting in cell injury or death. The latter can become particularly problematic in delicate tissues or when high vector doses are required, leading to the development of antibodies against the viral carrier. (d) Formation of antibodies: The antibodies against the viral carrier might be formed to cope with the past treatment, and this will reduce the effectiveness of the vectors the next time they are used. This can complicate the long-term therapies. (e) Inappropriate tumour proliferation in cancer gene therapy. In cancer gene therapy, which uses a virus to target and kill cancer cells, there is a risk of off-target effects and oncogene activation, neither of which is known to cause tumours. (f) Low ex vivo viability. Gene therapy requires specialised equipment and protocols for preparing and storing VVs ex vivo (2023). Table 2 compares viral and non-viral vectors with respect to payload capacity, immunogenicity, duration of expression, manufacturing complexity, and clinical applications.

**Table 2:** Comparison between viral and non-viral vectors

Parameter	Viral vectors	Non-viral vectors
Payload capacity	Limited; constrained by viral capsid size, often under 10 kb for some viruses, restricting large gene delivery	Generally higher; can accommodate larger genes or nucleic acids due to flexible design of carriers such as lipids and polymers (Zu and Gao 2021; Wang et al. 2013)
Immunogenicity	Typically higher; host immune responses to viral proteins can cause toxicity and neutralization, limiting re-administration (Baker et al. 2005; Honrath et al. 2025)	Lower; minimal immunogenicity due to synthetic materials such as lipids, polymers, and inorganic particles, contributing to better safety profiles (Ren et al. 2021; Zu and Gao 2021)
Duration of expression	Generally long-lasting; viral vectors can mediate stable and prolonged gene expression, sometimes permanent integration	Usually transient; non-viral vectors often show shorter expression durations, from hours to a few weeks, depending on nucleic acid type and formulation. For example, RNA lipid nanoparticles may express protein as quickly as 1 h but last only 5–7 h, while DNA lipoplexes may sustain expression for several weeks (Hauck and Hecker 2022)
Manufacturing complexity	Complex and costly; virus production requires cell cultures, purification, and stringent safety testing, making scale-up challenging (Zu and Gao 2021; Honrath et al. 2025)	Simpler and scalable; synthetic routes enable cost-effective production with fewer biosafety concerns, facilitating large-scale manufacture (Wang et al. 2025)
Clinical applications	Widely used in clinical gene therapies, including genetic diseases, cancer, and vaccine development; often selected when long-term expression is needed (Wang et al. 2025)	Increasingly applied in gene therapy and drug delivery, particularly for transient expression needs such as mRNA vaccines, CNS gene delivery, and cases requiring low immunogenicity. Active targeting strategies enhance specificity (Zu and Gao 2021; Hauck and Hecker 2022)

Viral vectors are capable of providing effective and long-term gene expression but suffer from a few drawbacks, such as immunogenicity, small payload capacity, and difficult manufacturing. Non-viral vectors are more secure, flexible, and scalable options thanks to their higher capacity; however, they offer temporary gene expression and still have to address the problem of transfection efficiency. Different non-viral systems, including lipid nanoparticles and polymer carriers, have recently emerged, which help break the barriers specific to certain clinical situations.

## Non-viral vectors

The non-viral gene delivery procedures, in their turn, include the tedious insertion of a piece of DNA into the cell by synthetic or natural compounds or physical forces. The viral ones are not as toxic or immunogenic as the media suggests. Moreover, cell- or tissue-specific models can be realised by utilising cell-specific functionality to develop chemical or biological vectors, whereas spatial control can be achieved through physical processes. The second possible advantage of nonviral techniques is that they are easy to manufacture and can be reused multiple times. Nonviral strategies are often considered less efficient than viral ones, and gene expression is rarely sustained in the long term. However, as reported more recently, certain physical systems for delivering genes have also become as efficient and can achieve the same expression time, which is clinically relevant (Al-Dosari and Gao 2009).

### Physical gene delivery methods

#### Needle and jet injection

The injection of naked DNA was first localised intramuscularly in 1990 and then in various other tissues, such as the brain, skin, and liver. DNA uptake is largely concentrated in the region of the needle track, implying that the uptake of DNA is caused partially by the physical damage caused by the insertion of the needle (Wolff et al. 1990; Freeman and Niven 1996; Glasspool-Malone and Malone 1999; Sato et al. 2000; Desigaux et al. 2005). Therapeutic gene direct injection into muscle or skin has been useful for assessing diverse factors related to DNA-based vaccination. Plasmid DNA encoding interleukin-12 and the carcinoembryonic antigen gene increased antitumor immunity in intramuscular injection (Song et al. 2000). Some promising results have been reported from a Phase I/II clinical trial evaluating the therapeutic effectiveness of direct myocardial injection of the Vascular Endothelial Growth Factor Receptor-2 (VEGF-2) gene in patients with severe ischemic heart disease, including improvements in exercise capacity (Losordo et al. 2002). By March 2009, over 18.1% (*n* = 278) of clinical trials in GT used the method. The jet injection initially was defined in 1947 when it was described as a no-needle technique of drug delivery as opposed to the traditional needle injection (Wendell et al. 2006). A high-speed, ultrafine stream of DNA solution is achieved using pressurised gas, typically Carbon Dioxide (CO2). The injection creates holes in the membranes of target cells through which intracellular gene transfer occurs. Depending on the mechanical properties of the target tissues, the degree of penetration power in this approach can be regulated by the gas pressure. The typical protocol for jet injection is to inject a DNA solution (1–5 µl is typical), set the injection pressure (1–3 bars is typical), aim the injector directly at the target tissue, and, lastly, press the trigger. Gene transfer has been performed using several jet injectors that differ in injection volume and repetitive injection mode. A head-on comparison of needle injection and different formulations of jet injectors indicated that the magnitude of gene expression under jet injection is 50 times more than that of traditional needle injection (Ren et al. 2002). Low-volume injector models are preferable to high-volume models when making multiple injections. The target tissues tolerate the transfer of the jet injection genes well, and no serious side effects have been reported. Yet, it has been claimed to cause localised pain, oedema, and bleeding at the injection site, especially when the old models of injectors were used (Lysakowski et al. 2003). Stein et al. have shown that following an in vivo intratumoral transfer of a short hairpin RNA expression vector targeting the multidrug resistance gene 1 (MDR1), a complete reversal of the MDR1 phenotype and, subsequently, improved chemotherapy to inhibit tumour growth were achieved (Stein et al. 2008). Additionally, jet injection is currently undergoing a Phase I clinical trial to treat skin metastases from malignant melanoma and breast cancer, and it has demonstrated in vivo efficacy for gene delivery (Walther et al. 2008).

#### Hydrodynamic gene transfer

It was described in a hydrodynamic procedure in 1999 (Liu et al. 1999). Upon rapid injection of a large volume of DNA solution into a mouse, transfection was effectively done in the lung, liver, heart and kidney, resulting in expression. The hydrodynamic technique uses high pressure to transfer the energy of a gene. When a large volume of DNA (approximately 8–12 per cent of body weight) is injected over a short period (3–5 s), endothelial permeability may change reversibly. Transient pores may form in the hepatocyte membranes, permitting the diffusion of DNA molecules into the hepatocyte (Zhang et al. 2004). Transfection of hepatocytes can be achieved effectively at 30–40 per cent. This is the most effective nonviral gene transfer technique for delivering genes in vivo in rodents. With this method, transgene expression was comparable to the physiological expression of genes. Catheter-assisted gene transfer via perfusion can also be successfully performed in the kidney, muscle, or a section of the liver (Eastman et al. 2002). The simplicity and safety of hydrodynamic gene delivery are that the concept of administering this procedure to deliver in vivo human genes was entirely ruled out, since a comparatively large injection would be required, which is well beyond acceptable levels in patients. Some modifications have been made to make this process less intrusive. For instance, effective gene transfer to large animals has been enabled by using a balloon catheter to introduce the catheter into the targeted tissue’s vasculature with reduced injection volume and speed (Al-Dosari et al. 2006; Suda and Liu 2007; Fabre et al. 2008). This suggests that hydrodynamic gene transfer can be applied in clinical settings, particularly given the recent development of a computer-controlled injector (Suda et al. 2008).

#### Gene gun

Gene therapy using gene guns, also called ballistic DNA transfer or DNA-coated particle bombardment, began in 1987, when genes were transferred into plants (Klein et al. 1987). This technology uses the properties of heavy metals on target tissues and demonstrates the process of loading coated DNA onto a particle as it passes by. Helium (the most commonly used inert gas) is pressurised to prepare particles for high-velocity firing into tissues. Gene guns have successfully delivered gene constructs to animal and plant cell culture tissue using large gold, tungsten or silver macroparticles. Factors such as gas pressure, particle size, and dosing frequency affect tissue penetration efficiency, tissue damage from the gun, and total levels of gene transfer (Uchida et al. 2002). Gene transfer with the gene gun has been tested extensively intramuscularly, intradermally and intratumorally for immunogenetics. The gene gun may elicit a vigorous immune response at lower doses than needle delivery in both large-animal models and human clinical trials. It has also been shown that vaccination in an animal model, now known to be a late-stage preclinical type 1 diabetes model with the GAD gene, promotes type 2 immunity, resulting in suppression of B-cell autoimmunity (Goudy et al. 2008).

Gene transfer with gene guns involves firing micro-projectiles made of tungsten or gold with DNA or RNA coating. The micro-projectiles are propelled by helium gas or some other propellant to get through cell membranes and inject genetic material into the cytoplasm or nucleus. In addition, while gene gun technology has hardly been used in clinical gene therapy of some vector-borne infections (malaria, dengue, Zika), this technology could be successfully implemented in vaccine delivery in the following sphere: Vaccine Administration with Gene Guns: One of the areas where gene guns have been used with success is vaccine administration for such diseases as malaria and dengue (Nishikawa and Huang 2001).

Cancer Gene Therapy: A noteworthy example is illustrated by the murine tumor models, where immunomodulatory gene delivery using gene gun technology such as IL-12 was instrumental when it came to tumor suppression. For example, administration of IL-12 gene via gene gun technique on mouse tumors transfected either in vitro or in the vaccination form had a significant impact on tumor suppression, thus proving the relevance of the choice of gene and expression dynamics (Kitagawa et al. 2003). Transient High-Level Expression in Immunotherapy: The gene gun provides a transitory but sufficient level of gene expression which is needed for the generation of an antitumor immune response, and does not present any risk of integration of viral vectors. Pulmonary and Respiratory Diseases: While not pure examples of gene gun technology applications, case studies of application of non-viral nanoparticles and vectors targeting lung epithelium with the use of integrin ligands may open up new possibilities for the future of respiratory gene therapy, which may not be limited to gene gun technologies only (Jenkins et al. 2000).

#### Electroporation

Since the 1960s, it has been known that electric fields can alter the permeability of cells. The first in vitro and in vivo applications of electroporation to introduce genes into cells were reported in 1982 and 1991, respectively. In vivo electroporation uses a series of electrical pulses to create temporary openings in the cell membrane, enabling electrophoresis-driven transport of DNA into cells (Neumann et al. 1982; Titomirov et al. 1991). For this approach, a DNA solution is injected into the target tissue, and a series of electrical pulses with varying voltage, pulse duration, and pulse number is applied using two electrodes. This approach is regarded as safe and effective compared with other non-viral delivery methods and is highly reproducible. The transfection efficiency of in vivo electroporation can be optimised to equal that of vaccination-elicited immunity (Andre and Mir 2004). In addition to local DNA injections for electroporation, Sakai et al. demonstrated that in vivo electroporation can also be applied locally after systemic injection of plasmid DNA. They reported localised transfection of a rat liver lobe following systemic administration of plasmid DNA, but little or no transfection in the adjacent lobes (Sakai et al. 2005). Marti et al. reported one of the first positive reports on in vivo electroporation and a potential new application for improving wound healing through electroporation-mediated delivery of growth factor-1 to the damaged area using keratinocytes as vector cells. This is an exciting advance, but the potential of electroporation for in vivo gene transfer to solid tissues is limited by the difficulty of reaching internal organs with electrodes. Continued research and improvement of fundamental technologies are likely to increase the potential for clinical applications of this technology (Marti et al. 2004).

A randomized controlled trial of phase I/II that was performed in Vietnam examines how beneficial and safe umbilical cord-derived mesenchymal stem cells (UC-MSCs) are when looked at in the context of treatment of frailty in older people. Comprehensive mechanistic analysis, including results of immune markers, as well as studying the cellular senescence process, is part of this project that allows tracking the therapeutic action of MSCs in this chronic condition. The results of the trial will be analyzed with a focus on adverse events that can occur during the trial as well as on the changes in frailty of older participants, while the research will look at the findings connected with secretions of cytokines and growth factors by MSCs in relation to clinical outcomes (Hoang et al. 2022).

Following the promising results of preclinical trials involving [177Lu]Lu-AKIR001, a CD44v6-targeted radiotherapeutic antibody, good tumor selectivity, radiodosimetry, and safety profiles were recorded in xenograft and animal models. Tumor regression occurred in a dose-dependent manner, with complete tumor regression observed in 80% of radioresistant models following fractionated dosing. Additional safety studies based on good laboratory practice promote its use in early clinical trials. A phase I clinical trial (NCT06639191), which is now actively recruiting, marks a rare transition from preclinical research on radionuclides to intervention in human solid tumors (Mortensen et al. 2026). mRNA vaccines using lipid nanoparticles (LNPs) completely reinvented the approaches to the prevention of infectious diseases and cancer immunotherapy. Studies in the realms of clinical trials and the real world provide evidence for its safety, immunogenicity, and efficiency while revealing some of the adverse events, such as myocarditis and inflammation of blood vessels. Continuous improvements conducted in means of delivery and personalized approach are aiming to prevent those complications and fully benefit from the capabilities of the technology. This systemic evaluation informs the subsequent improvements of vaccine development in terms of safety and immune response modification (Batisani and Chisompola 2026).

Some mAbs were associated with favourable safety profiles; however, they failed to achieve key clinical objectives, such as lowering the risk of pneumonia. The inconsistency of the findings may be due to the impact of biofilm formation and variability in the patients’ immune response. Future studies should focus on early intervention with mAbs, as well as optimizing doses and using mAbs in combination with other therapies (Piscaglia et al. 2025). Clinical trials of folate receptor-directed therapies in patients suffering from cancers that overexpress folate receptors have shown improved progression-free survival and response rates compared to conventional chemotherapy. The methods used in the studies, including folate-drug conjugates and folate-targeted nanoparticles, provided tumor-targeted effects with minimal toxicity. These studies show great potential of receptor-targeted delivery for achieving the desired therapeutic results with minimal side effects (Saraei et al. 2025).

#### Magnetofection

It relies on the theory of magnetically assisted drug delivery. The process depends on the conjugation of a therapeutic gene to a magnetic nanoparticle. This complex is realised in the cell culture. The rare-earth electromagnets inserted beneath the cell culture create a field gradient that increases the sedimentation rate of complex cells and improves transfection efficiency. Magnetic particles and therapeutic gene complexes are intravenously injected in vivo. Powerful external high-gradient magnets retain the complex on the target. This is the extrusion of genetic material due to degradation of a matrix, charge interactions or enzymatic cleavage of a cross-linking molecule. Its application is primarily in vitro research, and it is used to transfect primary cells and hard-to-transfect cells (Dobson 2006; Al-Dosari and Gao 2009; Jones et al. 2013).

Neural Stem Cell Therapy for Neurological Sequelae: Vector-borne illnesses like Zika virus and malaria are capable of inducing neurologic damage. By means of magnetofection, genetically engineered neural stem cells (NSCs) carrying minicircle DNA vectors were shown to exhibit high non-viral transfection rates (~ 54%) and maintain steady expression for weeks, helping to improve possible therapy for cerebrospinal damage due to vector-based diseases. This method is useful for overcoming the restrictions that conventional plasmids have and allows achieving better effectiveness in cell-based treatment of the CNS (Fernandes and Chari 2016). Vaccine Antigen Delivery Improvement: Magnetofection helps deliver DNA vaccines effectively, which is necessary for fighting against vector-borne viruses such as dengue fever and chikungunya. As magnetofection can help to localize complex vectors in mucosa or skin areas, which are under pressure from viruses using less active components, the approach can help to increase the stimulating potential of the vaccine (Laurent et al. 2011).

Magnetofection has demonstrated more efficient use in the gene delivery of non-vector diseases, including gene therapy for cancer, pulmonary gene therapy, and neurodegenerative illnesses: (a) Cancer Gene Therapy: Using magnetic particles along with viral and non-viral delivery systems has enhanced gene delivery efficiency by hundreds of times in vitro, with successful functionality tested in animal tumor models. Magnetic targeting has permitted local transfection in the bloodstream and intestines, suggesting intriguing possibilities for delivering oncogenes precisely or performing immunotherapy (Scherer et al. 2002). (b) Pulmonary Gene Delivery: Human respiratory epithelium is complicated because of mucociliary clearance. Research demonstrated that magnetofection significantly improves transfection efficiency in permanent and primary airways and ex vivo respiratory epithelium by enabling the complex to bind to the cell surface in a very short time and avoid the barrier of clearance (Gersting et al. 2004). c)Neural Stem Cell Engineering: Magnet-assisted transfection used in combination with multifection (multiple magnetofection procedures) resulted in better gene delivery to NSCs in monolayer and neurosphere in vitro cultures, which could be highly applicable in regenerative neurotechnology and treatment of diseases such as Parkinson’s disease and related neurodegenerative disorders (Pickard et al. 2017).

### Chemical-based nonviral vectors

Non-VVs based on chemicals may contain viruses, both natural and artificial. Chemical vectors are typically divided into categories: peptide-based, lipid-based, inorganic particles, and polymer-based. Overall, there are chemical non-viral nucleic acid delivery systems that comprise DNA/cationic lipid, DNA/cationic polymer, and DNA/cationic polymer/cationic lipid (Midoux et al. 2009).

#### Inorganic nanoparticles

The organic nanoparticles are traditionally composed of metals, inorganic salts, or ceramics (Sokolova and Epple 2008). The complexes formed by the metal ions in the salts are 10–100 nm in size. The surface coating of these nanoparticles can be used to enhance DNA binding or deliver genes to a specific location. The small particle size also offers several advantages, including the ability to circumvent most physiological and cellular barriers, thereby increasing gene expression (Cai et al. 2008). Moreover, they may cross the cellular wall via a specific membrane receptor or nucleolin, enabling cellular transfer of nanoparticles to the nucleus and avoiding endosomal-lysosomal degradation (Davis and Cooper 2007). Postmitotic cells can be effectively transfected using in vivo and in vitro nanoparticles. They will also most likely be non-toxic or least toxic and antigenic. Superparamagnetic iron oxide nanoparticles can provide magnetic responsiveness to an applied magnetic field and magnetically targeted delivery. Inorganic nanoparticle development for in vivo applications has taken massive momentum over the last few years. However, extensive research is required to determine the effects of their types, sizes, and shapes on transfection efficiency. It is undisputable that conducting additional studies can hasten their clinical applications, with special focus on long-term safety and further surface functionalization (Dong 2008).

#### Synthetic/Natural biodegradable

##### Cationic lipids

Hundreds of lipids have been invented to transfer genes. They all lack a variable structure except for a positively charged hydrophilic head, a hydrophobic tail, and a linker between the two. The neutral positively charged head group, which is located at the C-terminus, entraps the negatively charged phosphate group of the nucleic acid and leads to the formation of distinctly compacted lipoplexes. The general geometrical form, the number of charged groups per molecule, the type of lipid anchor, and the bonding of the molecule linker also rely on the efficiency of transfection. Its positive charge enables it to interact with the negatively charged glycoproteins and proteoglycans of the cell membrane via electrostatic interactions and to increase nucleic acid uptake. The lipids that cover the genetic material help protect it against intracellular and extracellular nucleases. However, the key is the surface charge, which reduces lipoplex half-life in circulation and decreases their capacity to cross vascular endothelial cells. A neutral polymer, e.g., polyethene glycol (PEG), helps to increase half-life and overcome excessive charging to achieve surface shielding. Lipid nanoemulsions are treated as low-toxicity lipoplexes; nevertheless, when the ratio of the lipid to the DNA is greater than 3:1, the lipoplexes become cytotoxic (Glover et al. 2005; Al-Dosari and Gao 2009; Lee and Lee 2012; Su et al. 2012; Gascón et al. 2013; Jones et al. 2013).

##### Lipid nanoemulsions

These are particles about 200 nm in size, composed of oil, water, and a surfactant. Lipid nanoemulsions are said to be better than liposomes in terms of scalability and stability, thereby becoming serum resistant. The data reveal that they are less toxic compared to liposomes (Al-Dosari and Gao 2009). Nanoemulsions are a kind of non-viral vector with great prospects for gene delivery in diseases where both vectors and no vectors are involved because they are biocompatible, produced easily, and able to carry genetic material effectively. Due to their unique structure, they have a possibility of using cationic lipids to be integrated into them, which can help in compacting and protecting nucleic acids and thus help in taking their molecules into cells and delivering the gene. One pertinent study illustrating the effectiveness of lipid nanoemulsions in gene delivery is described in the paper discussing the application of stearylamine-based nanoemulsions for DNA compaction. Stearylamine is a cationic lipid possessing primary amine groups, and it is located at the interface of oil and water in nanoemulsions, imparting positive charge to particles and thus allowing for electrostatic binding to negatively charged DNA molecules. Electrostatic binding is a necessary precondition for DNA compaction, preventing DNA degradation and improving intracellular delivery. The authors studied physical differences affecting the process, such as ways of incorporating stearylamine into nanoemulsions (in water or oil), incubation time, or temperature. The results showed that stearylamine incorporation in the aqueous phase led to the best DNA compaction that took place after 120 min of complexation and at low temperature (4 ± 1 °C). It is worth noting that the formation of lipoplexes resulted in a slight decrease in zeta potential of particles compared to nanoemulsions; however, the particle size did not change, which suggests that lipoplexes possess the necessary physicochemical properties for gene delivery (Silva et al. 2012).

Apart from the compaction and protection of genetic material, lipid-based nanocarrier systems have been investigated in targeted gene therapy for various disease conditions, including cancer and heart disease. Nanostructured lipid carriers (NLC) are a type of lipid nanoemulsion undergoing modification by incorporating targeting ligands, resulting in more specific and efficient transfection. For instance, in the case of lung cancer gene therapy, transferrin (Tf)-conjugated NLC was prepared with plasmid DNA encoding enhanced green fluorescence protein (pEGFP), showing remarkably higher efficiency in all cell lines tested (in vitro A549 cell line) and mouse models with A549 tumors bearing mouse models (in vivo). Tf ligand was responsible for its effectiveness in terms of receptor-mediated endocytosis, leading to the increased nuclear uptake of the genetic material. The size of the aforementioned Tf-NLC/pEGFP compound particle was more than 157 nm and contained a high amount of genetic material of more than 82%. These results indicate the flexibility of lipid-based nanoemulsions and their derivatives in designing the effective delivery system for gene treatment of non-vector disease like cancer (Han et al. 2016).

With regard to vector diseases, it should be noted that while there are no specific examples mentioned concerning the utilization of lipid nanoemulsions in the context of vector-borne diseases, the overall field of non-viral nanotechnology-based vectors utilizes a wide range of strategies such as lipid nanoparticles, liposomes, and lipid drug conjugates which are very widely used in the delivery of genetic materials for the cure of genetic disorders and different infectious diseases. Lipid nanoparticles have many positive features, like being biocompatible and producing them on a large scale; moreover, they can be sterilized and have stable delivery profiles. In addition, lipid nanoparticles interact with biological surfaces and cellular membranes much better, therefore such nanoparticles allow the delivery of drugs and genes to target organs (del Pozo-Rodríguez et al. 2013). Moreover, the implementation of non-viral vectors like lipid nanoemulsions into clinical practice is moving forward despite problems such as poor transfection efficacy and toxicity. New developments in nanotechnology seek to improve the physical and chemical parameters of nanocarriers, enabling them to tackle intracellular obstacles and thus improve treatment results for different kinds of diseases (Mirón-Barroso et al. 2021; Sainz-Ramos et al. 2021).

##### Solid lipid nanoparticles

Nanoparticles that are directed to solid lipid particles are made using lipids at both room temperature and body temperature. It possesses the advantages of cationic lipids and lipid nanoemulsions. It is shown that nucleic acids can be effectively stored in cationic solid lipid nanoparticles, thereby reducing nuclease-mediated degradation. It is currently being adopted as the delivery system of choice for siRNA (Al-Dosari and Gao 2009). An important piece of research investigated cationic solid lipid nanoparticles (SLNs), which were made using microemulsion technology and were complexed with plasmid DNA. These nanoparticles were positively charged (+ 44 mV) and were roughly 340 nm in size, which allowed them to form successful lipoplexes with DNA. Their characterization using scanning electron microscopy and differential scanning calorimetry demonstrated their efficacy and stability as non-viral vectors for gene delivery. Thus, SLNs proved their ability to compress and transport the genetic material, which is crucial for gene therapy (Carrillo et al. 2013). The advancements in the SLN technology include the development of PEGylated SLNs containing ionizable lipids (PEG-iSLMs) for efficient delivery of mRNA and plasmid DNA. By adjusting the ratios of the ionizable lipid (ALC-0315) and cationic lipid (DOTMA), PEG-iSLNs provided higher transfection capability and membrane fusion activity than formulations that contained only one type of lipid. In stability tests, it was established that these nanoparticles could provide desirable outcomes for a long time (Maniyamgama et al. 2025).

The SLNs were improved with modifications that enhanced targeting and transfection efficiency. For example, the SLNs were adapted with folate-modified dexamethasone for targeting tumor cells via receptor-mediated endocytosis. With this modification, SLNs were designed to improve the transport of genetic materials inside cells utilizing nuclear localization signals. The modified SLNs had a size of about 258 nm and were characterized by high values of gene loading (90%) and comparatively high transfection efficiency (~ 40% as opposed to 24% for unmodified SLNs) when applied to carcinoma cells in vivo and vitro. The low levels of cytotoxicity indicate that SLNs are biocompatible at therapeutic concentrations, thus being a good candidate for targeted non-viral gene delivery systems (Wang et al. 2014). SLNs continue to be studied as carriers of anticancer drugs. For example, SLNs loaded with tamoxifen that were produced through high-pressure homogenization showed proper characteristics for parenteral administration and demonstrated anti-proliferative activity close to that of free tamoxifen in laboratory studies against breast cancer cells. Thus, SLNs possess strong potential for utilization in targeted drug delivery within cancer therapies (ALHaj et al. 2008).

Additionally, SLNs have also been tested for DNA vaccine delivery. In a particular study, SLNs prepared with a solvent-emulsification method were conjugated with plasmid DNA carrying a gene for enhanced green fluorescent protein (eGFP) and tested for dendritic cells delivery. Such DNA-SLNs showed better transfection effectiveness than plasmid DNA alone and equal effectiveness as lipofectamine. The microscopy experiments confirmed the mechanisms of cellular uptake and endosomal escape that were important for gene delivery. The toxicity experiments showed very good biocompatibility, confirming the suitability of SLNs for the non-viral application of DNA vaccines (Penumarthi et al. 2017).

##### Peptide-based

It has been concluded that peptide-based vectors have superior capabilities compared to other non-VVs for compacting and protecting DNA in tight spaces, exhibiting cell receptor specificity, disrupting endosomal membranes, and delivering genetic cargo into the nucleus. Basic residues are found in high concentration in cationic peptides, including lysine and/or arginine. A particular site can be attacked by vectors when the vectors are bound to peptide ligands that bind to polyplexes or lipoplexes. Shorter peptide sequences derived from viral proteins help the vector deliver the nuclear localisation signal, aiding the transport of genetic material into the nucleus. As a result of these advantages, non-VVs have shown both strengths and weaknesses in functionalizing cationic lipoplexes or polyplexes (Al-Dosari and Gao 2009). The advantages of non-VVs in gene therapy include safety. They are said to be safer than the VVs, less likely to cause immune responses, and less cytotoxic and mutagenic. The versatility strength is another one. The advantages of using non-VVs include the delivery of a wide range of genetic cargo, such as plasmid DNA, mRNA, and small interfering RNA (siRNA). c) Non-VVs also reduce the chances of inadvertent genetic modification, which is an issue with certain VVs. The main advantage of non-VV gene therapies is that they can reduce production costs and produce therapeutics on a large scale, as the problem of producing large biomass does not arise in the production of viral-based products. In some applications, their use may be restricted by reduced transfection efficiency. Another drawback of non-VVs is that they do not produce a sustained effect on gene expression; hence, they require repeated treatment. Vectors are also prone to complications when used with certain cell types, limiting their use in some forms of gene therapy (Agarwal 2025). Viral and non-viral systems: Comparative analysis. Viral and non-viral systems are vital approaches for gene therapy because they facilitate the delivery of genetic material to target cells. There are two types of vectors: non-viral and viral. Both have strengths and weaknesses that will determine whether they are applicable in clinical as well as research practice. (Ramamoorth and Narvekar 2015; Bulcha et al. 2021; Butt et al. 2022; Taghdiri and Mussolino 2024).

## CRISPR-based gene delivery

Innovations in gene therapy and the implementation of CRISPR-based gene delivery are changing the way gene therapy is perceived. The rapid changes in technologies happen at an unforeseen pace and carry numerous difficulties related to their utilization in terms of deliverability, safety, efficacy, and consequences in practice. The analysis establishes a variety of methods of delivery, new inventions in the field, restrictions, and emerging ways of clinical use. CRISPR contains parts that have to be transferred effectively into cells to successfully inject genes. The methods of insertion can be divided into viral vectors and non-viral vectors, both of which possess their advantages and disadvantages. Viral vectors: Recombinant adeno-associated virus (rAAV) vectors are the most popular agents for delivering CRISPR mechanisms, mainly thanks to their being highly specific to the tissues, being able to support the activity of transgenes, and being safe to use in the process. However, the limited size of packaging of rAAV stands in the way to the successful use of CRISPR products and urges scientists to seek new solutions in the field and develop compact forms of Cas and dual or trans-splicing vectors (Uddin et al. 2020; Gil et al. 2026).

Non-Viral Vectors: The delivery of nanoparticles has become popular as it is a safe and versatile alternative. When compared with viral gene delivery approaches, nanoparticles provide controllable size, chemical properties, and low immune responses. Delivery of CRISPR components becomes possible with the help of lipid nanoparticles (LNPs) and polysaccharide-based systems and other functional nanocomposites (Sioson et al. 2021; Ghani et al. 2023; Manchanda et al. 2026). The novel delivery approaches include exosomes, which are biocompatible water-membrane vesicles able to carry CRISPR-Cas9 cargo. The usage of exosomes in gene delivery may help to deal with the issues of larger size and specific targeting of tissues (Aslan et al. 2024). Ocular gene therapy is one of the applications using nanoparticle technology that shows promise in overcoming the barriers of viral delivery and immunogenicity (Salman et al. 2022).

In the field of nanotechnology, there have been many breakthroughs that have allowed CRISPR delivery to be more efficient. Functional nanocomposites can combine various substances into a delivery system that can direct treatments toward the particular organ, leading to higher effectiveness with less negative side effects. An example of these processes involved patients participating in the phase I study where lipid nanoparticles were used for the delivery of CRISPR components (Ghani et al. 2023). Advances in the development of polysaccharide-based vectors allow solving safety and targeting problems and rely much on their natural compatibility and versatility to improve the delivery of CRISPR/Cas (Manchanda et al. 2026). The engineering of such CRISPR alterations, like ‘smaller’ Cas enzymes and such base editors, broadens the ability to deliver the therapeutic agent by using different delivery vectors, including viral tools needing a smaller size, and increases the accuracy of genome editing procedures (Mengistu 2026).

The full realization of CRISPR gene therapy is hampered by several significant hurdles. (a) Delivery Efficiency: There are issues with ensuring the proper delivery of CRISPR to the target cells. These include bypassing biological barriers and allowing effective cellular uptake and entry of the CRISPR components into the cell nucleus (Macarrón Palacios et al. 2024). (b) Immunogenicity: Therapy may fail or become hazardous due to the immune system reactions against the viral vectors and Cas proteins. Non-viral delivery systems may reduce, but it does not eliminate immunogenic concerns (Salman et al. 2022). (c) Off-Target Effects: Unintentional genetic changes still pose a serious risk. To prevent these effects, major advances in molecular design and delivery targeting have been made; however, evaluation of the long-term effects is still in progress (Toofan et al. 2026). (d) Cargo Size Restrictions: The bulky CRISPR systems will need new strategies, either using two viral vectors or compact enzyme systems for their successful delivery to cells because of the limitations imposed by viral vector capacity (Gil et al. 2026). (e) Heterogeneity of Editing: Editing efficiency can vary dramatically in different types of cells and tissues, which may create a challenge in obtaining desired therapeutic effects (Mengistu 2026). (f) Safety of Delivery Vehicles: The delivery vehicles (for instance, nanoparticles and vectors) may themselves show toxicity (Uddin et al. 2020; Salman et al. 2022).

CRISPR therapies have moved to the next phase of clinical trials for many genetic disorders, cancers, and viral infections. There are two types of therapy: ex vivo, which involves editing cells of patients before they are reintroduced back to the body, and in vivo, which works directly inside the body, and both are developing in their own way (Toofan et al. 2026). Ex vivo technique is helpful for the treatment of monogenic diseases since it leads to successful cell treatment without additional challenges posed by delivery (Salman et al. 2022; Macarrón Palacios et al. 2024; Gil et al. 2026). At the same time, the in vivo technique is making rapid progress in clinical use. Nanoparticle- and rAAV-mediated delivery is being researched now (Aslan et al. 2024).

The use of CRISPR for gene transfer has undergone a significant evolution since its early use of viruses, moving to new materials such as nano-composites, polysaccharides, and exosomes. Unfortunately, the success of the gene delivery system still remains second-rate due to low efficacy and immunogenicity. Recent work, however, indicates that improvement of the system is imminent. Initial clinical studies demonstrated successful evidence showing CRISPR’s efficacy in treating diseases, thereby confirming the necessity of future research in the area. Combining CRISPR molecular biology with state-of-the-art delivery systems has the potential to revolutionise medical sciences and provide new solutions in therapeutic applications.

## Innovation in vector development

Vector-based delivery has advanced significantly in recent years, particularly in vaccine delivery, gene therapy, and targeted drug delivery. Ensuring prolonged gene expression, preventing unfavourable immune responses, and maintaining effective, targeted delivery to the intended cells are among the many challenges associated with gene delivery. In addition to promoting advanced genetic research, these issues demand further studies to improve the safety, effectiveness, and precision of gene delivery technologies, which have significant potential to address genetic diseases and malignancies and to develop novel vaccines (Verma et al. 2024). Some of the novel technologies are discussed below:

### Engineering-enhanced targeting and delivery

To increase the efficacy and safety of novel approaches, such as CRISPR-Cas9 technology, addressing issues related to cell-type-specific distributions and reducing adverse effects throughout the delivery process are important (Aljabali et al. 2024). It can modify viral vectors to enhance site-specific, noninvasive gene delivery, thereby improving clinical outcomes (Li et al. 2024). Ligand-based targeting, surface modification, stimuli-responsive delivery and artificial intelligence (AI)-assisted targeting are some of the innovative strategies that are currently emerging.

In ligand-based vector engineering, vectors are modified by combining cell-specific peptides, antibodies, or antigens with aptamers (Reetz et al. 2014). This enhances receptor‒ligand interactions and accumulation, which reduces off-target toxicity (Dogbey et al. 2023). These ligands bind receptors, such as folate receptors and human epidermal growth factor receptor 2, that are overexpressed or concentrated on the cell surfaces of target cells, typically tumour cells (Prajapati et al. 2024).

Although transferrin is efficiently taken up by cells, this pathway can be used to deliver therapeutic genes, proteins, and chemotherapy drugs, primarily to rapidly growing cancer cells that overexpress transferrin receptors (Akhtar et al. 2014). To ensure appropriate drug distribution and specificity, capsids can be modified genetically or chemically to alter vector‒host interactions (Weklak et al. 2021). PEG coatings on nanoparticles prolong systemic circulation by protecting the nanoparticles’ surfaces against phagocytosis, opsonisation, aggregation, and other biological barriers (Suk et al. 2016).

Both exogenous (temperature, magnetic fields, and light) and endogenous (pH, redox, and enzymes) stimuli can elicit responses from stimulation-responsive viruses (Brun et al. 2017). Stimuli-responsive technology can improve therapeutic efficacy and avoid the side effects of parenteral or oral drug delivery by responding to particular triggers to release the therapeutic agent at the intended site (Rahim et al. 2021). Internal stimuli are the basis for pH- or redox-sensitive liposomes that release the drug in response to moderately acidic pH or GSH, respectively (Ashrafizadeh et al. 2022). Improvements in drug screening techniques are brought about by machine learning, deep learning algorithms, and AI-enhanced technologies (Singh et al. 2024). Vector modifications improve specificity, and computational models estimate the optimal design of tailored gene delivery vehicles (Varga et al. 2000).

### Hybrid systems

Various delivery methods for gene modification, such as viral and nonviral systems or combinations of both, can be found in hybrid systems. Researchers are working to develop hybrid vectors, viruses enclosed in chemical carriers. By encapsulating immune-modulating epitopes on viral vectors, these combination vectors have considerable potential to elude the host immune system. Molecules or scaffolds encapsulating viruses improve their capacity to target and release them over time to the intended target. A recently developed category of gene delivery vectors known as hybrid vectors, which combine chemical and viral vectors, overcomes the drawbacks of each vector while also enhancing desirable characteristics such as targeting ability, cytotoxicity, low immunogenicity, high payload, and the capacity to deliver multiple transgenes (Mahato et al. 2018). Recombinant viral hybrid vectors can incorporate their therapeutic DNA into donor genomes (Müther et al. 2009). Liposomes and lipid nanoparticles, single-chain cyclic polymers, highly branched poly(β-amino ester), polyethyleneimine, and poly(amidoamine) dendrimers are among the latest, most effective, and fastest-growing nonviral gene delivery vectors (Wang et al. 2023). Accurate and effective genetic changes in cells and tissues are enabled by combining liposome delivery systems with the CRISPR-based approach (Yin et al. 2023). Exosomes are tiny extracellular vesicles that, when associated with synthetic carriers or modified viral vectors, exhibit minimal immunogenicity and excellent biocompatibility, making them optimal drug delivery vehicles that can effectively target cells while reducing side effects and off-target adverse effects (Yin et al. 2023). Multifunctional nanoparticles offer a flexible framework for personalised medicine and have demonstrated significant promise across medical disciplines such as multimodal imaging, theranostics, and image-guided treatments (Bao et al. 2013).

### Novel materials and approaches

An additional opportunity to improve gene delivery effectiveness, optimise gene expression dynamics, regulate immune responses, encourage tissue regeneration, enhance stability, and enhance therapeutic efficacy involves incorporating innovative biomaterials into gene therapy techniques (Chen et al. 2023). Owing to their adaptable interactions with cells and biomolecules, peptides offer a unique opportunity to enhance the transport of nucleic acids (Urandur and Sullivan 2023). The development of effective drug delivery systems, molecular biology tools, and nanodevices for disease detection has long been a driving force behind nucleic acid nanoassembly research via DNA and RNA nanotechnology (Zhao et al. 2023). Current developments in the design and use of three DNA-based nanostructures include DNA hydrogels for RNA transport, lipid nanoparticle (LNP) technology associated with frame-guided assemblies (FGAs), and DNA origami (Wu et al. 2024). Three-dimensional bioprinting holds great promise, particularly when combined with microfluidic technologies, enabling more intricate, personalised, and networked models that could transform clinical applications and biomedical research (De Spirito et al. 2024).

## Clinical trials

The major clinical trials investigating gene therapy for both vector-borne and non-vector-borne diseases are given in Table 3.

**Table 3:** The major clinical trials investigating gene therapy for both vector-borne and non-vector-borne diseases

Disease /Indication	Vector type	Product/investigational name	Trial phase/status	Example trial number (registry ID)	Notes
β-Thalassemia (transfusion-dependent)	Lentiviral	Betibeglogene autotemcel (Zynteglo)	Pivotal Phase III → approved	NCT01745120	Autologous HSCs transduced with LV encoding β-globin; EMA/FDA approvals for TDT
Sickle cell disease	Lentiviral	Lovotibeglogene maraparvovec (Lyfgenia)	Phase II/III → approved	NCT02140554	Adds modified β-globin (anti-sickling); indicated for severe SCD with vaso-occlusive
Metachromatic leukodystrophy (MLD)	Lentiviral	Atidarsagene autotemcel (Libmeldy/Lenmeldy)	Phase II/III → approved	NCT01560182	LV-modified autologous HSCs express ARSA; used pre-symptomatically or early in disease.
Cerebral adrenoleukodystrophy (CALD)	Lentiviral	Elivaldogene autotemcel (Skysona)	Phase II/III → approved	NCT01896102	Alternative to allogeneic HSCT; improves neurologic outcomes in boys with CALD.
Severe combined immunodeficiency (ADA-SCID)	γ-retroviral/lentiviral	Strimvelis (γ-RV, EU) and newer LV programs	Phase I/II → approved (Strimvelis)	NCT01306019	Autologous CD34 + cells corrected for ADA; Strimvelis EMA-approved; LV approaches aim for improved safety.
Hemophilia B	AAV	Etranacogene dezaparvovec (Hemgenix)	Pivotal Phase III → approved	NCT03569891	Single-dose AAV5-FIX therapy; durable FIX expression with bleed reduction.
Hemophilia A	AAV	Valoctocogene roxaparvovec (Roctavian)	Phase III → approved	NCT03370913	AAV5-FVIII gene therapy with long-term factor expression; EMA/FDA approvals
Spinal muscular atrophy (SMA)	AAV9	Onasemnogene abeparvovec (Zolgensma)	Phase III → approved	NCT03306277	Systemic AAV9-SMN1 replacement for infants; transforms survival and motor outcomes
AADC deficiency	AAV2	Eladocagene exuparvovec (Upstaza)	Phase I/II → approved	NCT01302808	Direct CNS infusion improves motor function in an ultra-rare metabolic disorder.
Inherited retinal dystrophy (RPE65)	AAV2	Voretigene neparvovec (Luxturna)	Phase III → approved	NCT00999609	Restores functional RPE65 in biallelic RPE65 mutation; improves functional vision.
Melanoma (locally advanced)	Oncolytic HSV-1	Talimogene laherparepvec (Imlygic)	Phase III → approved	NCT00769704	Intralesional oncolytic virus expressing GM-CSF; improves durable response in melanoma.
hATTR amyloidosis (polyneuropathy)	LNP-siRNA	Patisiran (Onpattro)	Phase III → approved	NCT01960348	LNP-encapsulated siRNA targeting TTR; systemic knockdown.
hATTR amyloidosis (polyneuropathy)	GalNAc-siRNA	Vutrisiran (Amvuttra)	Phase III → approved	NCT03759379	Subcutaneous GalNAc-conjugated siRNA with infrequent

## Challenges and limitations of gene delivery

The challenges in gene delivery for disease vectors and non-vectors are diverse and range from extracellular to intracellular, and immunological to translational barriers in gene therapy for both viral and non-viral methods. To move forward in viral as well as non-viral gene therapy, it is necessary to understand the limitations involved in the gene delivery process, the common one being overcoming the biological barriers that take place between systemic therapy and gene expression at the intracellular level. Such obstacles include the breakdown of nucleic acids in the extracellular environment by nucleases, ineffective transportation to and uptake by target cells, retention in endosomal compartments, and barriers to nuclear access by DNA vectors. Naturally, viral vectors can evolve mechanisms that help in evading many of these barriers, and they manage to provide good transfection rates. However, these vectors have limited payload capacity, complicated production processes, and high levels of immunogenicity. On the contrary, non-viral vectors (lipid nanoparticles, polymers, inorganic nanoparticles, etc.) bring a number of advantages such as safety, low immunogenicity, ability to be scaled, and flexibility in payload, but their intracellular delivery effectiveness is unfortunately low, and so is the level of gene expression (Wang et al. 2025; Honrath et al. 2025).

Challenges in gene delivery stem from immune responses. Viral vectors can incite strong immune responses in their hosts so the duration of transgene expression is limited and vector can’t be re-administered and cause negative side effects. Despite being less immunogenic, non-viral vectors can also elicit an immune response based on their own components or on the molecules they deliver. For instance, plasmid DNA can be recognized by the immune system through the presence of CpG motifs, while siRNAs can trigger interferon response. All these immune activations lead to the complications of both safety and efficacy. The approaches to deal with the immune barriers include immunosuppression, the alteration of the route or dose administration, employing tissue-selective promoters, and modifying the surface of vectors. Yet, achieving a balance between killing the immune system and delivering the gene remains a complex task (Zhou et al. 2004; Hosseinkhani et al. 2015; Li et al. 2015). In addition, non-viral systems usually have lower efficiency than viral approaches, mainly due to the less effective intracellular dynamics and endosomal release. Some modern developments, such as ionizable lipids and multifunctional polymeric systems, tend to make a positive difference, but optimization is still necessary to obtain effective results in vivo. Speaking about manufacturing problems, scaling up production of viral vectors is complicated and expensive, which hinders widespread implementation of such vectors in clinical practice. In contrast to viral approaches, non-viral systems are cheaper and easier to produce, with good storability, but there are still problems with batch reproducibility and standardization for gene delivery systems (Wang et al. 2025; Honrath et al. 2025).

## Regulatory aspects of gene transfer technologies

Both viral and nonviral vector-based gene therapy are advancing for various health conditions. However, among these technologies, nonviral vector-associated gene transfer is growing tremendously compared with that of viral vectors. Between 2004 and 2013, the use of nonviral vectors in clinical trials increased as viral vector use significantly decreased. More nonviral vector drugs are now in clinical trials due to improvements in their efficiency, specificity, gene expression time, and safety (Ramamoorth and Narvekar 2015). The ethical, regulatory, and technical requirements for establishing clinical trials for gene therapies; the need for reviews or meetings with health authorities; the factors health authorities will consider in their scientific reviews; and the particular difficulties that must be overcome when developing gene therapy products in these areas. Finally, several strategic suggestions have been proposed, including streamlining the regulatory consultation process, improving the regulatory review system for several scientific assessments, and offering regulatory guidance and training initiatives to promote the development of gene therapy (Mizoguchi et al. 2024). However, there are safety, community-wide, and ethical issues with genetic engineering technology. These risks are particularly associated with genetically modified organisms (GMOs) and a rise in views that consider certain ethnic groups “more suitable” than others (FOUNDATION 2026). Research on gene therapy has advanced significantly over the last three decades, from simple laboratory tests to FDA-approved products that significantly reduce the disease burden for patients with severe health conditions (Lapteva et al. 2020). Gene transfer technologies are monitored, regulated and approved by international regulatory agencies worldwide.

The FDA regulates cellular and gene therapies (CGTs) in the US. Most of these therapeutic products are governed by the Office of Tissues and Advanced Therapies (OTAT) of the Centre for Biologics Evaluation and Research (CBER). Recent assessments show their eligibility to benefit from the knowledge of the Oncology Centre of Excellence (OCE) regarding cancer-related evidence. The Office of Vaccines Research & Review at CBER (OVRR) reviews therapeutic CGT medicines (Mendicino et al. 2019). The FDA regulates these products through Investigational New Drug Applications (INDs) and Biologics License Applications (BLAs). The FDA must approve an investigational drug or biological product before a clinical researcher is authorised to administer it to humans. The approval procedure is an investigational new drug application (Patel et al. 2026). A request for authorisation to market or ship a biologic product for entry into national/international trade is known as a biologics license application (BLA) (21 CFR 601.2) (Yang et al. 2026). The European Medicines Agency (EMA) Committee for Advanced Therapies (CAT) is the authority that monitors scientific advancements in therapeutic areas and evaluates the safety, efficacy, and quality of advanced therapy medical products (ATMPs) (López-Paniagua et al. 2021). ATMPs are divided into three primary categories: tissue-engineered, somatic-cell, and gene therapy medicines (Rejon-Parrilla et al. 2023). Other regulatory bodies, such as the Pharmaceuticals and Medical Devices Act of Pharmaceuticals and Medical Devices Agency (PMDA) Japan, the New Drugs and Clinical Trial Rules 2019 under Central Drugs Standard Control Organization (CDSCO) India and the National Medical Products Administration (NMPA) China, govern gene therapy regulations (Cornetta et al. 2022; Cai et al. 2024).

Regulatory agencies have developed various guidelines to standardise the approval process. The FDA has issued several important guidelines, including “Human Gene Therapy for Rare Diseases,” (2020) “Cellular and Gene Therapy Guidances,” “CMC Information for Human Gene Therapy Products final guidance 2020,” “Human Gene Therapy for Neurodegenerative Diseases final guidance 2022,” and “Long Term Follow-up after Administration of Human Gene Therapy Products Guidance for Industry January 2020” (FDA 2020).

The challenges and benefits of multiagency monitoring are illustrated by the evolution of gene transfer research-related regulations over time. Thus, the FDA and NIH have multiple roles, increasing confusion about the differences between protecting proprietary information and being transparent, between minimising adverse effects and promoting scientific advancement, and between setting standards and maintaining flexibility in a changing research market. Health technology assessment (HTA) departments lack frameworks or rules specific to gene therapy. In recent years, several gene therapies have been approved by international regulatory bodies and/or funded by reimbursement schemes. Limited information and uncertainty regarding cost-effectiveness, long-term safety, and efficacy are among the regulatory and reimbursement barriers (Viswanathan and Bubela 2015).

## Future perspective

Looking ahead, advancing gene delivery methods that make use of vectors and vectors alike points to the need for continuous refinement, innovation, and incorporation of state-of-the-art technologies in order to address existing issues while also amplifying the benefits of therapy. Viral vectors have been put at the centre of gene therapy since they are able to provide very high transduction rates and sustained gene expression. The future in the sector is believed to be focused on: (a) improving delivery method creation: Technologies such as hybrid delivery methods and capsid engineering based on artificial intelligence are aimed at improving the specificity of targeting, decreasing immune responses, and improving intracellular delivery of the genetic material. All these efforts will contribute to enhanced efficiency of the intervention along with minimized adverse effects; (b) implementation of CRISPR techniques: Although the implementation of CRISPR techniques into the methods of gene correction using viral vectors is expected to be used for the sake of precision medicine, the researchers will be able to create “custom-made” therapies for genetic and acquired diseases, as well as provide the possibility of regulating expression of transgenes; (c) development of relevant techniques.

Non-viral vectors have come to be acknowledged as safe and flexible, although they do not have a high level of transfection efficiency. New developments in: (a) Nanocarrier platform: Improvement on polymeric nanoparticles, lipid carriers, metal-organic frameworks, and engineered polymers is meant to increase uptake and targeting, as well as biocompatibility. (b) Smart delivery system: The emergence of multiple “smart” biomaterial scaffolds that are able to take those factors into account is expected to provide precise in situ delivery of genes. Their joint application with editing tools such as CRISPR/Cas9 allows local application in personalized therapy, thus getting rid of the donor cell requirement and immune rejection. (c) Combination therapy: Application of engineered non-viral vectors of various types for gene delivery together with medications for combination therapy.

In terms of neurological disorders, improvements in viral vectors, engineered microRNA, nanoparticles, and CRISPR-based therapies have made it possible to treat neurodegenerative diseases and unique genetic disorders. Future efforts will be geared towards optimizing delivery routes, creating more specific vectors, and finding new genes of interest. In the field of cancer gene therapy, personalized gene editing and immunotherapy techniques are changing the whole paradigm of treatment by reducing off-target activities and costs of procedures. The success achieved in the treatment of metabolic liver diseases has been reached with two gene delivery techniques, i.e. lipid nanoparticles and recombinant adeno-associated virus. Future research will be focused on overcoming immune activation and liver damage resulting from the usage of high doses of the vectors, as well as making the approaches more specific. In the field of hematopoietic stem cell therapy, the advances in viral transduction efficiency, together with the application of the small molecules that enhance the process of transduction and precision genome editing make it possible to treat hereditary hematologic disorders in a personalised manner.

Episomal vectors, known as efficient and ideal means to introduce therapeutic genes that do not confer the risks of insertion into a genome while providing stable extrachromosomal expression, thus leading to safer gene therapy methods, are becoming popular tools for the gene delivery industry. The combination of vector, as well as non-vector ways: The combination of viral and non-viral approaches, presumably in combination within hybrid vectors, could exploit the positive features of the modes of work through reaching both high efficiency and safety, thus increasing the efficacy of treatment. The transformation of gene application in vector-induced disease management: Although not conventional gene therapy, the genetic techniques that target the vector population together with the pathogen transmission patterns that rely on the population biology methods make it possible to develop new prevention measures against vector-borne diseases. In particular, the integration perspective is predominantly believed to imply uniting the genetically modified vectors with AI-optimised engineering solutions and gene editing technologies, as well as innovative non-viral techniques based on nanomaterials and biomaterial scaffolds, bringing together engineering developments aimed at overcoming biological barriers and helping to improve efficiency and safety.

## Conclusion

Gene transfer technologies have made monumental strides, revealing the important therapeutic promise of viral and non-viral vectors. Viral vectors, such as adeno-associated viruses and lentiviruses, continue to be preferred because of their high transfection efficiencies and the ability to achieve stable gene expression for long periods, which has made them useful in many clinical trials investigating genetic diseases and cancers. Conversely, non-viral methods provide advantages in terms of safety profiles, as they can minimize immunogenicity and allow for a much greater degree of customization, even in terms of scalability. On the downside, however, their transfection efficiencies are lower compared to those of viral vectors. Breakthroughs in the field of CRISPR-Cas technology have increased the accuracy and flexibility of these methods, bringing a new outlook on gene treatment in many fields, such as regenerative medicine and targeted therapy for cancer. Despite such progress, viral and non-viral systems face different problems; while in the case of viral vectors, it is the immune response and possible insertional mutagenesis, in the case of non-viral platforms, the problem is in the efficiency of delivery. The evolution of regulatory issues is seen to accompany the progress of technology, with active attempts to create comprehensive regulatory systems that guarantee the necessary level of safety, quality assurance, and ethical standards for clinical application of the technologies. The future appears to be focused on hybrid vectors combining the advantages of both systems, the development of transcending and efficient gene editing tools, and targeted delivery methods ensuring better specificity and efficiency. To sum up, continuous advancements in vector technology, as well as innovations in genome editing and delivery methods, should contribute to a broader range of therapeutic solutions. Solving the existing challenges will rely on a complex approach to innovations regarding the realisation of the potential for gene transfer technologies to be used in personalized medicine.

## Abbreviations

GT: Gene therapy
AIDS: Acquired immunodeficiency syndrome
AD: Alzheimer’s disease
VVs: Viral vector
ASOs: Atisense oligonucleotides
DNA: Deoxyribonucleic acid
TALENs: Transcription activator-like effector Nucleases
Ad2: Adenovirus 2
AAV: Adeno-associated virus
EMA: European medicines agency
HSVs: Herpes simplex viruses
VEGF-2: Vascular endothelial growth factor receptor
CO₂: Carbon dioxide
MDR1: Multidrug resistance gene 1
